# Nano-Lazar: Read across Predictions for Nanoparticle Toxicities with Calculated and Measured Properties

**DOI:** 10.3389/fphar.2017.00377

**Published:** 2017-06-16

**Authors:** Christoph Helma, Micha Rautenberg, Denis Gebele

**Affiliations:** In Silico Toxicology gmbhBasel, Switzerland

**Keywords:** nanoparticle, toxicity, QSAR, read-across, predictive toxicology, machine learning, k-nearest-neighbors

## Abstract

The lazar framework for read across predictions was expanded for the prediction of nanoparticle toxicities, and a new methodology for calculating nanoparticle descriptors from core and coating structures was implemented. Nano-lazar provides a flexible and reproducible framework for downloading data and ontologies from the open eNanoMapper infrastructure, developing and validating nanoparticle read across models, open-source code and a free graphical interface for nanoparticle read-across predictions. In this study we compare different nanoparticle descriptor sets and local regression algorithms. Sixty independent crossvalidation experiments were performed for the Net Cell Association endpoint of the Protein Corona dataset. The best RMSE and *r*^2^ results originated from models with protein corona descriptors and the weighted random forest algorithm, but their 95% prediction interval is significantly less accurate than for models with simpler descriptor sets (measured and calculated nanoparticle properties). The most accurate prediction intervals were obtained with measured nanoparticle properties (no statistical significant difference (*p* < 0.05) of RMSE and r^2^ values compared to protein corona descriptors). Calculated descriptors are interesting for cheap and fast high-throughput screening purposes. RMSE and prediction intervals of random forest models are comparable to protein corona models, but *r*^2^ values are significantly lower.

## 1. Introduction

Read across is a commonly used approach for the risk assessment of chemicals and has recently gained popularity for nanoparticle risk assessment (Arts et al., 2014). Read across procedures are based on the assumption that similar substances cause similar biological effects. In order to estimate the activity of a novel substance a researcher will search for similar substances with known biological activities and deduce the activity of the new substance from this data.

Most read across procedures for nanoparticles originate from a regulatory setting and aggregate current nanotoxicity knowledge into rules for determining groups of similar substances and rules for extrapolating the toxicity of the unknown nanoparticle (see Arts et al., 2014 for a review, Arts et al., 2015; Schultz et al., 2015; Dekkers et al., 2016 for recent proposals).

Despite their popularity current read across approaches have a couple of disadvantages, especially in respect to the reproducibility and validation of prediction results:

- They require a lot of time from skilled toxicologists to search for data, interpret it according to guidelines and to aggregate it into a final assessment.
- Grouping and extrapolation criteria are rarely formally defined and leaves the risk assessor room for interpretation.
- Implicit assumptions about grouping and extrapolation criteria have been rarely validated and may be correct or not
- It is hardly possible to validate the proposed schemes with independent test sets of statistically relevant size.

In order to make the read across procedure reproducible, traceable and objective the authors of this paper have developed a programming framework (lazar, Maunz et al., 2013) for small compounds with well defined structures. lazar follows the generic read across process of identifying similar substances and extrapolating from their measured activities, but automates the process with well defined user selectable algorithms (see below). This makes predictions less time consuming, reproducible and allows independent validation studies. A graphical user interface presents the rationales of predictions and supporting information for a critical inspection and to reject dubious predictions.

The objective of the current study was to extend lazar for the risk assessment of nanomaterials and to integrate it with databases and ontologies of the eNanoMapper EU FP7 project (Jeliazkova et al., 2015), which contains currently all *public* nanoparticle datasets and to validate a subset of the implemented algorithms. The nano-lazar extension implements new methods for representing and handling nanomaterials without well defined chemical structures. This includes e.g., nanoparticle characterizations by structural, size and shape, physico-chemical and biological properties as well as ontology terms. It provides also nanoparticle specific methods for descriptor calculation, feature selection, similarity calculation and a graphical interface optimized for nanoparticle predictions.

Similar to lazar, nano-lazar is completely modular. Modelers can choose from a broad range of algorithms for descriptors (measured and calculated), feature selection, similarity calculation and local (Q)SAR models, or easily add new developments.

The concept of chemical *similarity* is the key idea behind all read across procedures. But similarity is not an intrinsic property of substances, it can be defined in different ways and the utility and performance of similarity measures depends on each specific use case.

*Structural similarity* is most frequently used in the risk assessment of compounds with well defined chemical structures. Structural similarity definitions are obviously not directly applicable to nanomaterials, because they lack a well defined structure. It is however relatively straightforward to adapt other concepts, e.g., similarity in terms of chemical properties or in terms of biological effects. Compared to structural similarity, which can be calculated directly from chemical structures, these similarity definitions depend on actual measurements, which makes their estimation more expensive and time consuming. For this reason we have developed a new structural similarity concept for nanomaterials, which is based on chemical fingerprints of core and coating materials.

In order to estimate the utility of various similarity concepts for nanomaterials, we have performed model building and validation experiments for models based on

- *Structural similarity* (using core and coating fingerprints)
- *Property similarity* (using measured nanoparticle physico-chemical properties)
- *Biological similarity* (using serum protein interaction data)

and the local regression algorithms

- Weighted average
- Weighted partial least squares
- Weighted random forests.

In addition we intend to address the important topic of *reproducible research* with this publication. In our experience it is frequently impossible to reproduce computational experiments for a variety of reasons, e.g.,

- Publications lack important details about algorithms.
- Publications do not provide access to the data that has been used.
- Authors use proprietary software that does not disclose its algorithms with all necessary details.
- Original software, libraries and operating systems are outdated and not available anymore.

We try to address these problems by providing a public, self contained docker image with all software and data required for the experiments presented in this manuscript at DockerHub (https://hub.docker.com/r/insilicotox/nano-lazar-paper). It contains also a build system for the manuscript, that pulls results and figures directly from validation experiments (similar to the R knitr package, Xie, 2015). Apart from repeating the experiments for this paper this image can also be used for extending the system, testing other descriptor and modeling algorithms and comparing validation results with the current benchmark as well as for teaching purposes.

Source code for the manuscript and validation experiments has been published under a GPL3 license at GitHub (https://github.com/opentox/nano-lazar-paper). The lazar framework library has been published under the same license (https://github.com/opentox/lazar).

A graphical webinterface for nano-lazar model predictions and validation results is publicly accessible at https://nano-lazar.in-silico.ch, source code for the GUI can be obtained from https://github.com/enanomapper/nano-lazar.

GitHub and DockerHub repositories are tagged with nano-lazar-paper to identify the software version that corresponds to the published paper. As this project is under continuous development, it is likely that some of the algorithms will change in the future. In this case it is relatively straightforward to identify differences with the versioning system or to use the submitted version as benchmark for further developments.

## 2. Methods

The following sections give a high level overview about nano-lazar algorithms. Readers interested in unambiguous algorithm definitions should refer to the source code links in the text.

### 2.1. Datasets

Nanoparticle characterizations and toxicities were mirrored from the eNanoMapper database (Jeliazkova et al., 2015) via its REST API (https://github.com/opentox/lazar/blob/nano-lazar-paper.revision/lib/import.rb#L9-L122). At present only the *Net cell association* endpoint of the *Protein corona* dataset, has a sufficient number of examples (121) to create and validate read-across models, all other eNanoMapper toxicity endpoints have less than 20 examples, which makes them unsuitable for local QSAR modeling and crossvalidation experiments.

Net cell association indicates the fraction of nanoparticles associated with A549 human lung epithelial carcinoma cells, including internalization of the nanoparticles and adhesion to the cell membrane (Walkey et al., 2014). Net cell association was measured in by inductively coupled plasma-atomic emission spectroscopy (ICP-AES) in A549 cells, which are widely used as a model to study fundamental nanoparticle-cell inter- actions. Net cell association has a relevance to inflammatory responses, biodistribution, and toxicity *in vivo* (Walkey et al., 2014). During the rest of the text we will frequently use the general term *toxicity* to indicate *Net cell association*, in order to increase readability and to emphasize the general applicability of the nano-lazar approach.

### 2.2. Algorithms

For this study we have adapted the modular lazar (*la*zy *s*tructure *a*ctivity *r*elationships) read across framework (Maunz et al., 2013) for nanoparticle model development and validation.

lazar was originally developed for small molecules with a defined chemical structure and uses chemical fingerprints for the identification of similar compounds (*neighbors*). Most nanoparticles do not have clearly defined chemical structures, but they can be characterized by their composition (core and coatings), measured properties (e.g., size, shape, physicochemical properties) or the interaction with biological macromolecules. Within nano-lazar we use these properties for the identification of similar nanoparticles (*neighbors*) and as descriptors for local QSAR models.

nano-lazar makes read-across predictions with the following basic workflow: For a given nanoparticle lazar

- Searches in the database for similar nanoparticles (*neighbors*) with experimental toxicity data,
- builds a local QSAR model with these neighbors and
- uses this model to predict the activity of the query compound.

This procedure resembles an automated version of *read across* predictions in toxicology, in machine learning terms it would be classified as a *k-nearest-neighbor* algorithm (https://github.com/opentox/lazar/blob/nano-lazar-paper.revision/lib/model.rb#L191-L272).

Apart from this basic workflow nano-lazar is completely modular and allows the researcher to use arbitrary algorithms for similarity searches and local QSAR modeling. Within this study we are using and comparing the following algorithms:

#### 2.2.1. Nanoparticle descriptors

In order to find similar nanoparticles and to create local QSAR models it is necessary to characterize nanoparticles by descriptors. In this study we are using three types of descriptors:

**Structural descriptors:** Union of MOLPRINT 2D fingerprints (*MP2D*, Bender et al., 2004) for core and coating compounds (https://github.com/opentox/lazar/blob/nano-lazar-paper.revision/lib/nanoparticle.rb#L22-L29)
  MP2D fingerprints use atom environments as molecular representation, which resemble basically the chemical concept of functional groups. For each atom in a molecule it represents the chemical environment using the atom types of connected atoms. MP2D fingerprints were calculated with the OpenBabel (O'Boyle et al., 2011) library.
**Physico-chemical nanoparticle properties:** Measured nanoparticle properties from the eNanoMapper database (*P-CHEM*).
**Biological nanoparticle properties:** Protein interaction data from the eNanoMapper database (*Proteomics*).

Nanoparticle MP2D fingerprints are a novel development for the characterization of nanoparticles with well defined core and coating compounds. In this case it is possible to create molecular fingerprints for all of these compounds and to use the union of these fingerprints as nanoparticle fingerprint. Based on our experience with small molecules we have selected MP2D fingerprints (Bender et al., 2004), which typically outperform predefined fingerprints (e.g., *MACCS*, *FP*4) for QSAR purposes. Despite its simplicity the concept works surprisingly well (see validation results) and enables toxicity predictions without measured properties. This can be useful e.g., for fast and cheap nanoparticle toxicity screening programs.

#### 2.2.2. Feature selection

Calculated MP2D fingerprints are used without feature selection, as preliminary experiments have shown, that feature selection deteriorates the overall performance of fingerprint read-across models (which is in agreement with our observations on small molecules).

Nanoparticle properties in the eNanoMapper database have not been measured for the purpose of read across and QSAR modeling. For this reason the database contains a lot of features that are irrelevant for toxicity. In preliminary experiments we have observed that using all available features for similarity calculations leads to neighbor sets that are unsuitable for local QSAR models, because large numbers of irrelevant features override the impact of features that are indeed relevant for toxicity.

For this reason we use the lazar concept of *activity specific similarities* (Maunz et al., 2013), by selecting only those features that correlate with a particular toxicity endpoint (Pearson correlation p-value < 0.05). This reduced set of *relevant features* is used for similarity calculations and local QSAR models (https://github.com/opentox/lazar/blob/nano-lazar-paper.revision/lib/feature_selection.rb#L7-L34). Apart from being computationally cheaper, simple filter methods pose also a lower risk of overfitting than more aggressive feature selection methods (e.g., forward selection, backwards elimination). As local models are built with the Rcaret package which uses feature selection internally there is no requirement for extremely small descriptor sets at this stage.

For crossvalidation experiments feature selection is repeated separately for each crossvalidation fold, to avoid overfitted models (Gütlein et al., 2013).

#### 2.2.3. Neighbor identification

For binary features (MP2D fingerprints) we are using the union of core and coating fingerprints to calculate the Tanimoto/Jaccard index and a similarity threshold of *sim* > 0.1 (https://github.com/opentox/lazar/blob/nano-lazar-paper.revision/lib/similarity.rb#L22-L27).

For quantitative features (P-CHEM, Proteomics) we use the reduced set of relevant features to calculate the *weighted cosine similarity* of their scaled and centered relevant feature vectors, where the contribution of each feature is weighted by its Pearson correlation coefficient with the toxicity endpoint. A similarity threshold of *sim* > 0.5 was used for the identification of neighbors for local QSAR models (https://github.com/opentox/lazar/blob/nano-lazar-paper.revision/lib/similarity.rb#L50-L66).

In all cases nanoparticles that are identical to the query particle are eliminated from neighbors to obtain unbiased predictions in the presence of duplicates (https://github.com/opentox/lazar/blob/nano-lazar-paper.revision/lib/model.rb#L234-L255).

#### 2.2.4. Local QSAR models and predictions

For read-across predictions local QSAR models for a query nanoparticle are build from the set of similar nanoparticles (*neighbors*).

In this investigation we are comparing three local regression algorithms:

- Weighted local average (*WA*, https://github.com/opentox/lazar/blob/nano-lazar-paper.revision/lib/regression.rb#L7-L21)
- Weighted partial least squares regression (*PLS*, https://github.com/opentox/lazar/blob/nano-lazar-paper.revision/lib/caret.rb#L8-L86)
- Weighted random forests (*RF*, https://github.com/opentox/lazar/blob/nano-lazar-paper.revision/lib/caret.rb#L8-L86)

In all cases neighbor contributions are weighted by their similarity to the query particle. The weighted local average algorithm serves as a simple and fast benchmark algorithm, whereas partial least squares and random forests are known to work well for a variety of QSAR problems. Partial least squares and random forest models use the R package caret (Kuhn, 2008). Models are trained with default settings, optimizing the number of PLS components or number of variables available for splitting at each RF tree node by bootstrap resampling.

Finally the local model is applied to predict the activity of the query nanoparticle. The RMSE of bootstrapped model predictions is used to construct 95% prediction intervals at 1.96^*^RMSE (https://github.com/opentox/lazar/blob/nano-lazar-paper.revision/lib/caret.rb#L59-L71).

If PLS/RF modeling or prediction fails, the program resorts to using the weighted average method.

For the weighted average algorithm prediction intervals are not available, because weighted average does not use internal validation.

#### 2.2.5. Applicability domain

The applicability domain of lazar models is determined by the diversity of the training data. If no similar compounds are found in the training data (either because there are no similar nanoparticles or because similarities cannot be determined due to the lack of measured properties), no predictions will be generated. Warnings are also issued, if local QSAR model building or model predictions fail and the program has to resort to the weighted average algorithm (https://github.com/opentox/lazar/blob/nano-lazar-paper.revision/lib/model.rb#L191-L272).

Each prediction is accompanied with a list of neighbors and their similarities, which are clearly displayed in the graphical user interface for the inspection by a toxicological expert. Apart from indicating the applicability domain, the neighbor list clearly shows the rationale for the prediction, and allows the expert to reject predictions e.g., when neighbors act via different mechanisms.

The accuracy of local model predictions is indicated by the 95% prediction interval, which is derived from internal caret validation (https://github.com/opentox/lazar/blob/nano-lazar-paper.revision/lib/caret.rb#L59-L71). Query substances close to the applicability domain (many neighbors with high similarity) will have a narrower prediction interval than substances with a larger distance (few neighbors with low similarity).

#### 2.2.6. Validation

For validation purposes we use results from 5 repeated 10-fold crossvalidations with independent training/test set splits for each descriptor/algorithm combination (https://github.com/opentox/lazar/blob/nano-lazar-paper.revision/lib/crossvalidation.rb#L100-L113). Feature selection is performed for each validation fold separately to avoid overfitting. For the same reason we do not use a fixed random seed for training/test set splits. This leads to slightly different results for each repeated crossvalidation run, but it allows to estimate the variability of validation results due to random training/test splits.

In order to identify significant differences between validation results, outcomes (RMSE, *r*^2^, correct 95% prediction interval) are compared by ANOVA analysis, followed by Tukey multiple comparisons of means (https://github.com/enanomapper/nano-lazar-paper/blob/nano-lazar-paper.revision/scripts/cv-statistics.rb).

Please note that recreating validations (e.g., in the Docker image) will not lead to exactly the same results, because crossvalidation folds are created randomly to avoid overfitting for fixed training/test set splits.

These five 10-fold crossvalidations are assigned to the final model, which is build from the complete training data. This validated model is used for further predictions, e.g. from the graphical webinterface.

### 2.3. Availability

**Public webinterface:** https://nano-lazar.in-silico.ch
**lazar framework** https://github.com/opentox/lazar (source code)
**nano-lazar GUI:** https://github.com/enanomapper/nano-lazar (source code)
**Manuscript:** https://github.com/opentox/nano-lazar-paper (source code for the manuscript and validation experiments)
**Docker image:** https://hub.docker.com/r/insilicotox/nano-lazarpaper/ (container with manuscript, validation experiments, lazar libraries and third party dependencies).

## 3. Results

The *Protein corona dataset* contains 121 Gold and Silver particles that are characterized by physchem properties (*P-CHEM*) and their interaction with proteins in human serum (*Proteomics*). In addition *MP2D* fingerprints were calculated for core and coating compounds with defined chemical structures.

Five repeated crossvalidations with independent training/test set splits were performed for the descriptor classes

- *MP2D* fingerprints (calculated, binary)
- *P-CHEM* properties (measured, quantitative)
- *Proteomics* data (measured, quantitative)
- *P-CHEM* and *Proteomics* data combined (measured, quantitative)

and the local regression algorithms

- Local weighted average (*WA*)
- Local weighted partial least squares regression (*PLS*)
- Local weighted random forests (*RF*).

**Figure 1:**
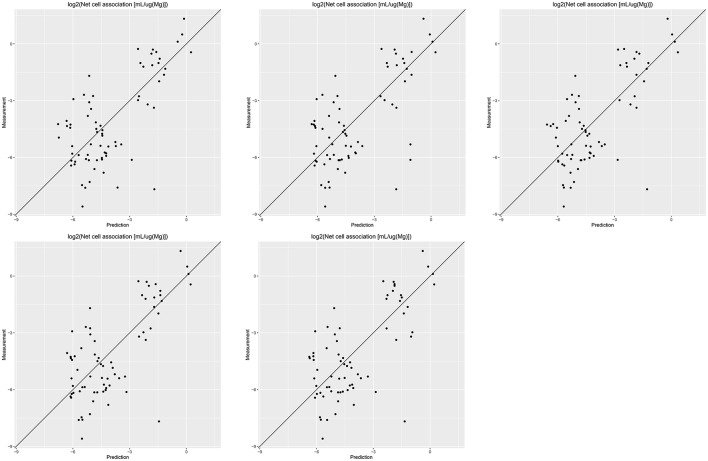
Correlation of predicted vs. measured values for five independent crossvalidations with *MP2D* fingerprint descriptors and local *random forest* models.

**Figure 2:**
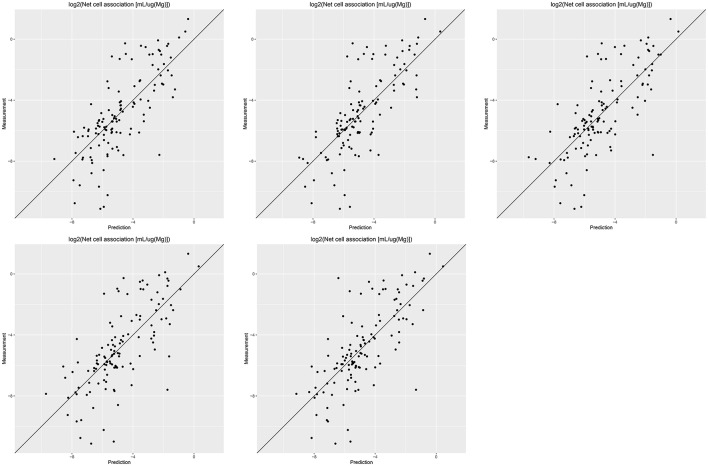
Correlation of predicted vs. measured values for five independent crossvalidations with *P-CHEM* descriptors and local *random forest* models.

**Figure 3:**
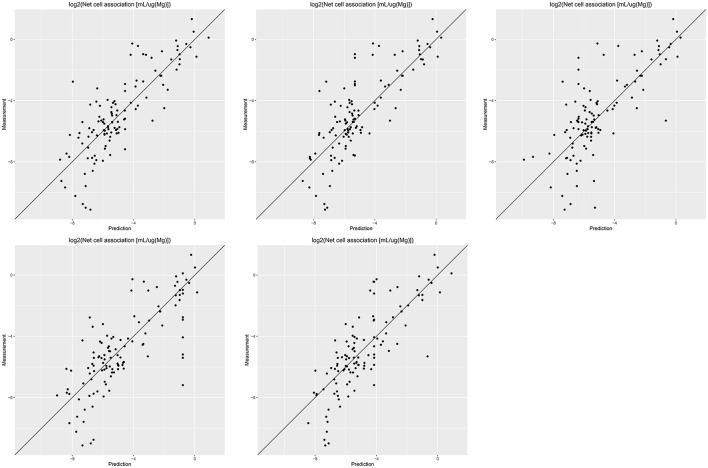
Correlation of predicted vs. measured values for five independent crossvalidations with *Proteomics* descriptors and local *random forest* models.

Results of these experiments are summarized in Table 1. Figures 1–3 show the correlation of predictions with measurements for *MP2D, P-CHEM*, and *Proteomics* random forests models. Correlation plots for all descriptors and algorithms are available as Supplementary Material (https://github.com/enanomapper/nano-lazar-paper/tree/nano-lazar-paper.revision/figures). Table 2 lists *P-CHEM* properties of the Protein Corona dataset and their correlation with the *Net Cell Association* endpoint.

**Table 1:** Results from five independent crossvalidations for various descriptor/algorithm combinations.

**Descriptors**	**Algorithm**	**RMSE**	***r^2^***	**% Measurements within prediction interval**
MP2D	WA	*2.03 2.1 2.07 2.07 2.03*	*0.24 0.19 0.21 0.22 0.24*	NA
MP2D	PLS	*2.05 2.03 2.02 2.09 2.16*	*0.28 0.28 0.29 0.27 0.28*	96 94 94 93 94
MP2D	RF	1.73 1.77 1.67 1.67 1.73	*0.46 0.45 0.49 0.5 0.47*	96 93 94 94 96
P-CHEM	WA	*1.98 1.94 1.91 1.93 2.0*	*0.44 0.47 0.48 0.47 0.43*	NA
P-CHEM	PLS	*2.09 2.09 2.14 2.03 2.01*	*0.38 0.39 0.36 0.42 0.43*	**97 96 97 96 97**
P-CHEM	RF	1.76 1.73 1.81 1.86 1.83	0.56 0.58 0.54 0.51 0.53	97 95 94 93 94
Proteomics	WA	1.88 1.72 1.73 1.91 1.76	0.52 0.6 0.59 0.52 0.58	NA
Proteomics	PLS	1.74 1.85 1.78 1.61 1.68	0.59 0.56 0.56 0.64 0.62	*87 87 86 85 88*
Proteomics	RF	**1.51 1.61 1.8 1.73 1.56**	**0.68 0.65 0.55 0.6 0.65**	*87 89 89 92 92*
P-CHEM Proteomics	WA	1.72 1.77 1.85 1.44 1.67	0.6 0.58 0.55 0.7 0.62	NA
P-CHEM Proteomics	PLS	1.55 1.91 1.79 1.94 1.64	0.67 0.54 0.58 0.51 0.64	*84 86 88 86 90*
P-CHEM Proteomics	RF	*1.85 1.74 2.1 1.68 1.51*	*0.55 0.59 0.45 0.61 0.69*	*90 88 90 91 92*

*Best results (mean of 5 crossvalidations) are indicated by bold letters, statistically significant (p < 0.05) different results by italics. Results in normal fonts do not differ significantly from best results*.

**Table 2:** *P-CHEM* properties of the *Protein corona* dataset measured with and without human serum.

**Property**	**Medium**	**Unit**
Localized Surface Plasmon Resonance (LSPR) index	–	
**Localized Surface Plasmon Resonance (LSPR) index**	**Human serum**	
LSPR peak position (nm)	–	nm
**Polydispersity index**	–	nm
Polydispersity index	Human serum	nm
**Core size**	–	nm
**Autot (ICP-AES)**	**Human serum**	nmol
Total surface area (SAtot)	Human serum	cm^2^
Protein density	Human serum	μg/cm^2^
**Total protein (BCA assay)**	**Human serum**	μg
**ZETA POTENTIAL**	–	mV
**ZETA POTENTIAL**	**Human serum**	mV
Z-Average Hydrodynamic Diameter	–	nm
**Z-Average Hydrodynamic Diameter**	**Human serum**	nm
Volume Mean Hydrodynamic Diameter	–	nm
**Volume Mean Hydrodynamic Diameter**	**Human serum**	nm
Number Mean Hydrodynamic Diameter	–	nm
Number Mean Hydrodynamic Diameter	Human serum	nm
Intensity Mean Hydrodynamic Diameter	–	nm
**Intensity Mean Hydrodynamic Diameter**	**Human serum**	nm

*Features correlating with the Net cell association endpoint (relevant features) are indicated by bold letters*.

Table 1 summarizes the results from five independent crossvalidations for all descriptor/algorithm combinations. The best results in terms of *RMSE* and *R*^2^ were obtained with *Proteomics* descriptors and local weighted *random forest* models. Six models have no statistically significant difference in terms of *RMSE* and five models in terms of *r*^2^. The most accurate 95% prediction intervals were obtained with *P-CHEM* descriptors and *partial least squares* models, these models does not differ significantly from the best *RMSE* and *r*^2^ results.

### 3.1. Descriptors

In terms of descriptors the best overall results were obtained with *Proteomics* descriptors. This is in agreement with previous findings from other groups (Walkey et al., 2014; Liu et al., 2015; Papa et al., 2016). It is however interesting to note that prediction intervals are significantly more inaccurate than those from other descriptors and the percentage of measurements within the prediction interval is usually lower than 90% instead of expected 95%.

Using *P-CHEM* descriptors in addition to *Proteomics* does not lead to improved models, instead we observe an increased sensitivity toward training/test set splits (crossvalidation variability) and *random forest* results perform even significantly poorer than *Proteomics* descriptors alone.

*P-CHEM* descriptors alone perform surprisingly well, especially in combination with local *random forest* models, which does not show statistically significant differences to the best *Proteomics* model. On average more than 95% of the measurements fall within the 95% prediction interval, with significantly better results than for *Proteomics* descriptors. A summary of *P-CHEM* descriptors can be found in Table 2.

All *MP2D* models have poorer performance in terms of *r*^2^, but the *random forest* model does not differ significantly in terms of *RMSE* and measurements within the prediction interval.

### 3.2. Algorithms

With the exception of *P-CHEM*/*Proteomics* descriptors *random forests* models perform better than *partial least squares* and *weighted average* models with significant differences for *MP2D* and *P-CHEM* descriptors (detailed pairwise comparisons are available in the Supplementary Material https://github.com/enanomapper/nano-lazar-paper/blob/nano-lazar-paper.revision/results/). Interestingly the simple *weighted average* algorithm shows no significant difference to the best performing model for the *Proteomics* and *P-CHEM*/*Proteomics* descriptors.

## 4. Discussion

### 4.1. Performance

Although *random forest* models with *Proteomics* descriptors have the best performance in terms of *RMSE* and *r*^2^, the accuracy of the 95% prediction interval is significantly lower than for *MP2D* and *P-CHEM* models (detailed pairwise comparisons in the Supplementary Material).

These problems seem to originate from internal caret optimisation and validation algorithms which underestimate *RMSE* values, that are used to calculate the prediction interval (see Algorithm section). The observation that the *weighted average* algorithm, which does not use caret, performs comparatively well for *Proteomics* descriptors, supports this interpretation.

Our initial suspicion was that an unfavorable ratio between descriptors (785 before feature selection, 129 after feature selection) and training examples (121) causes this problem. *Randomforest* and *partialleastsquares* algorithms are on the other hand robust against a large number of descriptors and caret returns very realistic *RMSE* values for *MP2D* fingerprints with a similar number of independent variables (100). For this reason it is presently still unclear, why prediction intervals for *Proteomics* descriptors are more inaccurate than for other descriptor types.

*P-CHEM random forest* models have the most accurate prediction interval and the *RMSE* and *r*^2^ performance is comparable to the *Proteomics* model, although they utilize a much lower number of descriptors (20 before feature selection, 10 after feature selection). The main advantage from a practical point of view is that predictions of novel nanoparticles require a much lower amount of measurements than with *Proteomics* data (although this argument may become obsolete with new high throughput techniques).

*MP2D* fingerprint descriptors are interesting from a practical point of view, because they do not require any measurements of nanoparticle properties. They need however defined chemical structures for core and coating compounds, which makes this approach infeasible for nanoparticle classes like carbon nanotubes. The resulting models do not differ significantly from the best results in terms of prediction accuracy (*RMSE*, measurements within prediction interval), but are significantly lower in terms of explained model variance (*r*^2^). For practical purposes one may argue that the primary objective of read across models is to make accurate predictions (low *RMSE*, accurate prediction interval) and not to explain the model variance (*r*^2^). For this reason we consider *r*^2^ performance as secondary compared to *RMSE* and prediction interval accuracies.

### 4.2. Problematic predictions

In order to investigate possible systematic errors with nano-lazar models we have investigated all *random forest* crossvalidation predictions with measurements outside of the 95% prediction interval.

Table 3 shows, that the number of problematic predictions increase from fingerprints to *P-CHEM* and *Proteomics* descriptors. Few substances have consistent incorrect predictions across all five crossvalidation runs, and it seems that models with Proteomics descriptors are more sensitive toward training/test set splits than e.g., fingerprint models. This observation is also supported by the poorer accuracy of their prediction intervals (Table 1).

**Table 3:** Random forest predictions with measurements outside of the 95% prediction interval (Median log2 transformed values).

**Descriptors**	**Nanoparticle**	**CVs**	**PI distance**	**Error**
MP2D fingerprints	G15.DDT@SDS	5	2.2	6.2
MP2D fingerprints	G15.NT@DCA	5	0.7	3.0
MP2D fingerprints	G60.MBA	5	0.5	2.7
MP2D fingerprints	G15.DDT@ODA	1	1.1	5.0
MP2D fingerprints	S40.MHDA	1	0.0	3.4
MP2D fingerprints	S40.CIT	1	0.0	2.3
MP2D fingerprints	G30.DDT@HDA	1	0.0	4.2
P-CHEM	G30.cPEG5K-SH	5	2.3	4.5
P-CHEM	G15.nPEG5K-SH	5	1.0	5.4
P-CHEM	G60.mPEG5K-SH	5	0.7	4.3
P-CHEM	S40.AUT	4	0.7	3.0
P-CHEM	G15.DDT@CTAB	3	0.9	6.1
P-CHEM	G15.HDA	2	0.3	5.6
P-CHEM	S40.PLL-SH	2	0.1	2.2
P-CHEM	G15.PEI-SH	1	0.5	4.6
P-CHEM	G15.DDT@SA	1	0.4	1.2
P-CHEM	G60.DTNB	1	0.2	1.7
P-CHEM	G15.MES	1	0.2	2.3
P-CHEM	S40.MAA	1	0.1	2.6
P-CHEM	G60.MBA	1	0.0	1.6
Proteomics	G15.nPEG5K-SH	5	1.3	3.9
Proteomics	G15.mPEG1K-SH	5	0.8	3.5
Proteomics	G30.cPEG5K-SH	5	0.6	3.9
Proteomics	G15.ODA	4	1.8	4.5
Proteomics	G60.NT@PVA	4	0.3	2.8
Proteomics	G60.MUTA	4	0.3	1.5
Proteomics	G30.AUT	4	0.2	0.6
Proteomics	G30.CALNN	3	0.3	2.1
Proteomics	G15.PEI-SH	3	0.3	0.3
Proteomics	S40.AUT	2	1.6	3.3
Proteomics	G60.mPEG5K-SH	2	0.9	2.9
Proteomics	S40.LA	2	0.1	1.3
Proteomics	G60.HDA	1	2.4	3.7
Proteomics	G15.MES	1	1.8	3.2
Proteomics	G15.PEG3K(NH2)-SH	1	1.8	3.9
Proteomics	G60.ODA	1	1.0	4.2
Proteomics	G15.AUT	1	0.1	0.4
Proteomics	G15.SA	1	0.1	0.8
Proteomics	G60.CIT	1	0.1	0.7
P-CHEM and Proteomics	G15.ODA	5	2.0	5.0
P-CHEM and Proteomics	G15.mPEG1K-SH	5	0.8	3.1
P-CHEM and Proteomics	G30.CALNN	5	0.7	2.2
P-CHEM and Proteomics	G15.nPEG5K-SH	5	0.6	3.4
P-CHEM and Proteomics	G60.MUTA	5	0.5	1.5
P-CHEM and Proteomics	G60.DTNB	4	1.1	1.6
P-CHEM and Proteomics	S40.AUT	3	1.6	3.3
P-CHEM and Proteomics	G60.mPEG5K-SH	2	0.4	3.5
P-CHEM and Proteomics	G30.AUT	2	0.3	0.8
P-CHEM and Proteomics	G15.AUT	2	0.1	0.4
P-CHEM and Proteomics	G15.MUA	2	0.1	1.1
P-CHEM and Proteomics	G30.cPEG5K-SH	1	2.4	3.5
P-CHEM and Proteomics	G15.PEG3K(NH2)-SH	1	1.2	2.8
P-CHEM and Proteomics	G15.PEI-SH	1	0.3	0.3
P-CHEM and Proteomics	G15.HDA	1	0.2	3.9
P-CHEM and Proteomics	G15.DDT@ODA	1	0.1	2.0
P-CHEM and Proteomics	G15.SA	1	0.1	0.7
P-CHEM and Proteomics	G15.PVA	1	0.0	1.7

Fingerprint models seem to provide the most stable predictions, but three nanoparticles have consistent problematic predictions across all crossvalidations. For illustrative purposes we will investigate G15.DDT@SDS, the substance with the largest prediction error.

In all five crossvalidations the closest neighbors (S40.DDT@DOTAP, G30.DDT@DOTAP, G15.DDT@DOTAP, G60.DDT@DOTAP) have a similarity of 0.5 and measured values between −2.0 and −0.3. This explains, why local models cannot extrapolate to the measured value of −7.7 of the query particle. Based on our experience with small molecules, we do not expect reliable predictions, unless local models can be built with a similarity threshold of 0.5^1^. Predictions obtained from neighbors with lower similarities can still be useful, but require manual inspection (and possible rejection) of a toxicological expert. For this purpose we provide the free graphical user interface at https://nano-lazar.in-silico.ch, which presents prediction results, neighbors and supporting information (e.g., links to additional eNanoMapper data, nanoparticle characterizations and ontologies).

### 4.3. Comparison with other models

According to our knowledge up to now no validated read across models have been published for the Protein corona datasets. Most other nanoparticle read across models have not been formally validated, with the exception of Gajewicz et al. (2015) and Gajewicz et al. (2017), who validated read across models for 17 metal oxides. Results from these studies are not comparable with our findings, because they use a different, smaller dataset and other validation methods. It seems that in both studies feature selection was performed on the complete dataset prior to model validation, which transfers information from the test set into the validation model. Single training (*n* = 10) and test (*n* = 7) sets were used, which makes it hard to ensure that models are not overfitted for the particular training/test set split. Due to the small test set size it is also hard to draw general conclusions about the model performance. We are not aware of any nanoparticle read across validation that exceeds 100 substances as in our investigation.

For the Protein corona dataset a couple of QSAR studies with global models have been published (Walkey et al., 2014; Liu et al., 2015; Papa et al., 2016), but unfortunately their results are also not directly comparable, because we report results for the complete dataset with 121 Gold and Silver particles, while other authors report results only for a subset of Gold particles.

Walkey et al. (2014) report leave-one-out (*LOO*) and 4-fold crossvalidation (4*CV*) results for 105 Gold particles. They obtained the best results (LOO *r*^2^ 0.86, 4CV *r*^2^ 0.63) with partial least squares models, protein corona data with four additional physicochemical parameters and jackknife parameter selection. Parameter selection was performed by crossvalidation, but it is unclear if parameters were selected on the complete dataset prior to LOO/4CV or separately for each LOO/4CV model. Performance wise the findings are roughly in agreement with our results. Assuming that feature selection was performed within crossvalidation folds we would expect 10-fold crossvalidation results between *LOO* and 4*CV* results. According to the authors the model developed for Gold compounds have little predictivity for Silver compounds, but a separate Silver model gave LOO *r*^2^ of 0.79. *RMSE* values are not available, although they are in our opinion more relevant for the predictive toxicology use case than *r*^2^ values (prediction error vs explained model variance).

Liu et al. (2015) report a 4CV *r*^2^ of 0.843 for 84 Gold compounds using ϵ-support vector machines (ϵ-SVM) with 6 serum proteins and zeta potential as descriptors. Descriptors were selected with sequential forward floating selection (*SFFS*). The methodological descriptions do not indicate explicitly, if feature selection was performed on the complete dataset or within 4CV folds. Judging from Figure 2 of this paper and the Methods section we have the strong impression that feature selection was performed prior to crossvalidation, which increases the likelihood of overfitted models, especially for aggressive feature selection schemes like *SFFS*. The 4CV *r*2 of 0.843 is clearly higher than our results, but it remains unclear, if the superior performance is due to better algorithms, a smaller more “regression friendly” dataset or overfitted models. Again we would have preferred *RMSE* values for comparison purposes, which are unfortunately not available.

Papa et al. (2016) developed global models for 84 Gold compounds with eleven algorithms and reported *r*^2^ and *RMSE* values for training set retrofitting, leave-one-out crossvalidation (*LOO*) and stratified external test set predictions (64 particles training set, 20 particles test set). There was little difference between good performing models (PPR, EARTH, SVM-linear, SVM-radial, MLR, and PLS) and the authors conclude that Projection Pursuit Regression (PPR) gives the most robust models (LOO *r*^2^ 0.81, *RMSE* 1.01, external *r*^2^ 0.79, *RMSE* 1.01). Feature selection (with genetic algorithms and support vector machines) and parameter selection (with the caret R package) were correctly performed on the training set only, which might explain the lower *r*^2^ values compared to Liu et al. (2015). Both *r*^2^ and *RMSE* values are better than in our study, but we have used the complete dataset with 121 Gold and Silver compounds and not a subset of 84 Gold compounds.

All these studies use global models for a subset of the Protein Corona dataset, which makes sense for a relatively homogeneous dataset with a single mode of action. nano-lazar in contrast creates local QSAR models for each query compound, which makes the approach more generally applicable for nanoparticles with different modes of action. For this reason we were able to cover all 121 nanomaterials of the Protein Corona dataset, while global models could utilize only 69% of the complete dataset. According to our experience with small molecules, local read across models are best applied to heterogeneous datasets with a couple of hundred examples. Datasets with approximately 100 examples are the lower margin where local QSAR models can be successfully built and validated. For this reason we expect that nano-lazar performance will increase as soon as more nanotoxicity data becomes available.

## 5. Conclusion

We have performed 60 independent crossvalidation experiments for the Protein Corona dataset obtained from the eNanoMapper database in order to identify the best combination of descriptors for nanoparticle read across predictions. The best RMSE and *r*^2^ results were obtained with protein corona descriptors and the weighted random forest algorithm, but the 95% prediction interval is significantly less accurate than that of models with simpler descriptor sets (measured and calculated nanoparticle properties). The most accurate prediction intervals were obtained with measured nanoparticle properties with RMSE and *r*^2^ values that show no statistical significant difference (*p* < 0.05) to the protein corona descriptors. Calculated descriptors are interesting for cheap and fast high-throughput screening purposes, they have significantly lower *r*^2^ values than the best results, but RMSE and prediction intervals show no significant difference to the best results of our investigation.

For practical purposes we suggest to use nanoparticle properties when measurements are available and the newly developed nanoparticle fingerprints for screening purposes without physicochemical measurements. Both models have been implemented with a graphical user interface which is publicly available at https://nano-lazar.in-silico.ch.

## Author contributions

CH was responsible for the design and implementation of the nano-lazar libraries, the validation studies and the text of this manuscript. DG and MR participated as scientific programmers in the development of nano-lazar libraries and in the validation experiments. They are the authors of the nano-lazar GUI and REST interfaces and contributed to the manuscript with critical revisions and proofreading.

### Conflict of interest statement

The authors declare that the research was conducted in the absence of any commercial or financial relationships that could be construed as a potential conflict of interest.

## Footnotes

^1^The latest lazar development version already issues a warning in this case, this feature will be included into the next release.
